# Russian bank data: Reasons of bank closure

**DOI:** 10.1016/j.dib.2020.105343

**Published:** 2020-02-28

**Authors:** Alexei Karas

**Affiliations:** a Utrecht University, University College Roosevelt, Lange Noordstraat 1, 4331 CB, Middelburg, the Netherlands; b Utrecht University School of Economics, the Netherlands

**Keywords:** Russia, Bank data, Bank failure, Bank default

## Abstract

For every closed Russian bank I collect its closure reasons. I divide these reasons into categories and report them in the form of tags. The data come from online sources. Combined with Russian banks' financial statements, freely available on the Central Bank of Russia's website, this dataset provides ample possibilities for empirical research: on bank failure prediction models that take into account the after-the-fact revealed reasons of bank closure; on the extent, determinants, and effects of financial misreporting by Russian banks; on the relative performance of banks pursuing particular business models, such as captive banks.

**Table:** Specifications Table

Subject	Finance
Specific subject area	Empirical Banking in Russia
Type of data	Table (Excel Format)
How data were acquired	Hand collected from public online sources
Data format	Raw
Parameters for data collection	Closure reasons of Russian banks
Description of data collection	Hand collected from public online sources
Data source location	Russia
Data accessibility	Repository name: Mendeley Data DOI: https://doi.org/10.17632/g7msxkcg55.2 URL: https://data.mendeley.com/datasets/g7msxkcg55/2

**Table:** 

**Value of the Data** • These data report closure reasons of Russian banks. • The data benefits researchers who study Russian banks or compare banking systems across countries. • The data can be used to empirically develop failure prediction models that take into account the after-the-fact revealed reasons of bank closure. • The data can be used to study financial misreporting and money laundering by Russian banks.

## 1 Data

The dataset [1] represents a table in Excel. Variables are in columns; variable names are in the first row. Rows correspond to different observational units, that is, different Russian credit institutions. Each institution is identified by its unique official registration number reported in the first column. This registration number should be used as key when merging the dataset with other data on Russian banks [2]. The list of institutions is limited to those that have (been) closed down. The closure date is reported in the second column. In what follows I use the words ‘bank’ and ‘credit institution’ interchangeably.

For each bank the third column reports a list of 5-character tags separated by spaces. Each tag corresponds to a specific reason of bank closure. Missing values represent banks for which closure reasons are not available. The next section provides further details.

## 2 Experimental design, materials, and methods

When a bank closes down the Central Bank of Russia (CBR) issues an official communication in which it explains the reasons of closure. Excerpts from these official communications are reported on a number of online portals. In what follows, I draw on the 2700+ excerpts reported in the Memory Book of Banki.ru [3].

I read the excerpts. As I read, I encounter reasons of bank closure. For each newly encountered reason I generate a tag, which I then place next to every bank whose excerpt contains that reason. For example, a tag *S1mac* is placed next to every bank that closed down because of a merger or acquisition. Banks with multiple reasons get multiple tags.

The correspondence between tags and closure reasons is summarized in Table 1. The first column reports the tags. The second column reports the reasons of closure. The third column reports the most common phrases the Central bank uses in its official communications that correspond to those reasons. All tags are divided into 8 categories. I discuss these categories below.

**Table 1:** Tag description.

Tag	Reason	Central Bank mentions
A1llr	insufficient provisions for losses	underestimates risk; overestimates asset quality; creates insufficient loss reserves
A2rsk	too much risk	risky credit policy; excessive risk; low asset quality
C1fin	poor financial condition	unsatisfactory financial condition; hides real financial condition; unsuccessful attempts to improve the situation; large asset loss, etc.
C2sol	solvency risk	bankrupt; need for measures to avoid bankruptcy; complete loss of capital; insolvent
C3c2p	insufficient capital adequacy	capital adequacy critically low/below two percent
C4low	insufficient capital	capital lower than required
E1neg	loss-making	suffers losses; negative net income
L1deb	debit balance	debit balance on correspondent account with the CBR
L2lbl	default on obligations	unable to/does not honor its obligations (in time)
M1cap	captive	captive bank; serving business interests of owners/managers
M2shd	dubious activities	dubious, shadow, doubtful, fictitious, transit operations/activities
M3tun	asset tunneling	asset tunneling; replaces liquid assets with illiquid
M4frd	fraud	fraud; financial pyramid; fake
M5ter	violates anti-money laundering law	violations of the federal law “On Counteracting the Legalization of criminal proceeds and the Financing of Terrorism” or related regulations
R1fed	violates laws	violates federal banking laws
R2cbr	violates CBR regulations	violates CBR regulations
R3nrm	violates prudential standards	violates prudential standards for statutory ratios (norms)
R4req	violates rules on reserve requirements	violates rules on reserve requirements
R5act	no activity	no activity/transactions; delay in starting operations
R6lic	lack of appropriate license	executing transactions without appropriate license
R7stc	violates statutory capital formation rules	violates rules for statutory capital formation
S1mac	MA	merger; acquisition; transformation into a subsidiary
S2liq	voluntary liquidation	voluntary liquidation
T1mis	financial statements unreliable	unreliable financial statements; violates principles of accounting; misreporting; factual absence of assets; etc.
T2non	financial statements lacking	did not submit financial statements
T3aud	no audit	no audit
T4ddl	financial statements submitted late	misses deadlines for submitting financial statements; delayed submissions

A-tags concern asset risk. *A1llr* tags banks that create insufficient provisions for possible losses. The CBR reports these banks to underestimate risk, overestimate asset quality, and create insufficient loss reserves. *A2rsk* tags banks that take too much risk. Their excerpts mention high risk credit policy, excessive risk taking, low asset quality, large amount of distressed assets, and similar. The two A-tags often co-occur, that is, banks that take excessive risk tend to underprovision for it.

C-tags concern capitalization and solvency. *C1fin* tags banks whose financial health the CBR considers generally unsatisfactory. This dissatisfaction is revealed in statements like ‘unsatisfactory financial condition’, ‘hides real financial condition’, ‘fails to improve the situation’, ‘experiences problems’, and other pejorative statements. The reasons for CBR's dissatisfaction are often unspecified. *C2sol* tags banks that are (on the verge of being) insolvent. The CBR reports these banks to be insolvent, bankrupt, in need of measures to avoid bankruptcy, or having lost all of their capital. *C3c2p* tags banks with too low capital adequacy. Their excerpts mention capital adequacy being critically low or below two percent. *C4low* tags banks whose capital falls short of established requirements, most frequently, short of established minimum value of statutory capital. The CBR often identifies these banks as failing to bring capital in line with requirements.

*E1neg* tags loss-making banks, that is, banks with negative net income.

L-tags concern liquidity. *L1deb* tags banks whose excerpts mention ‘debit balance on correspondent account (with the CBR)’. This ‘debit balance’ represents a form of borrowing from the Central bank. It was prohibited in 1995. *L2lbl* tags banks that have (partly) defaulted on their obligations. The CBR identifies these banks as being unable to honor, or effectively not honoring, (some of) their obligations.

M-tags concern specific aspects of the bank's business model. *M1cap* tags captive banks. The CBR reports these banks to primarily serve the business interests of (i.e. lend to) a narrow group of customers connected to the bank's owners (or managers). *M2shd* tags banks engaged in dubious activities. Their excerpts mention dubious, shadow, doubtful, fictitious, transit (and other synonyms of shady) operations/activities or examples thereof. *M3tun* tags banks whose excerpts mention asset tunneling, for example, in the form of replacing liquid assets with illiquid. *M4frd* tags banks whose closure relates to some sort of fraud. Their excerpts mention ‘fraud’, ‘financial pyramid’, and ‘fake’. *M5ter* tags banks whose excerpts mention violations of the anti-money laundering law (i.e. the federal law “On Counteracting the Legalization of criminal proceeds and the Financing of Terrorism”) or related regulations.

R-tags concern violations of rules and regulations (with the exception of anti-money laundering law violations). *R1fed* tags banks that violate federal banking laws. *R2cbr* tags banks that violate CBR regulations. *R3nrm* tags banks that violate prudential standards for statutory ratios (norms). *R4req* tags banks that violate rules on reserve requirements. *R5act* tags banks that got a license but did not start operations. Their excerpts mention ‘no activity’, ‘no transactions’, and ‘delay in starting operations’. *R6lic* tags banks that engaged in certain activities without having the appropriate license. *R7stc* tags banks that did not follow the right procedure when paying their initial capital. The CBR reports these banks to have violated rules for statutory capital formation.

T-tags concern transparency. *T1mis* tags banks engaged in financial misreporting. The CBR reports these banks to submit unreliable financial statements, to violate principles of accounting, to misreport specific balance sheet items, and similar. Note that insufficient provisions for possible losses are also a form of misreporting. For that reason, all banks tagged with *A1llr* should be considered as also tagged with *T1mis*. *T2non* tags banks that did not submit their financial statements to the CBR. *T3aud* tags banks that did not undergo a yearly audit. *T4ddl* tags banks that did not submit their financial statements on time.

*S1mac* tags banks that discontinue operations because of a merger, acquisition or transformation into a subsidiary. These banks come in two varieties. Some remain healthy until merger or acquisition. Others experience problems and are subject to Central Bank intervention. For these latter banks their acquisition is often facilitated by the Central Bank. Unfortunately, my data do not allow to distinguish these two varieties. Finally, *S2liq* tags banks that discontinue operations because of a voluntary liquidation.
